# When AI threatens future careers: a dual-appraisal model of university students’ career goal adjustment intentions

**DOI:** 10.3389/fpsyg.2026.1879844

**Published:** 2026-07-08

**Authors:** Leilei Zhang, Fangfang Long, Dan Chen

**Affiliations:** 1 School of Airline Service and Tourism Management, Guilin University of Aerospace Technology, Guilin, China; 2 Office of Student Affairs, Guilin University of Aerospace Technology, Guilin, China

**Keywords:** AI job replacement threat, career goal adjustment, cognitive appraisal theory, university student employment, proactive personality

## Abstract

**Introduction:**

Artificial intelligence (AI) is reshaping future work and increasing university students’ concerns about career uncertainty and employability. Existing research offers limited insight into why students respond differently to the same AI-related career uncertainty. Drawing on cognitive appraisal theory, this study examines how the AI job replacement threat influences students’ career goal adjustment intentions through challenge and hindrance appraisals, as well as whether proactive personality shapes these appraisal processes.

**Methods:**

Two studies were conducted. Study 1 used a scenario-based experiment with 205 university students to manipulate high versus low AI job replacement threat. Independent-samples *t*-tests and partial least squares structural equation modeling (PLS-SEM) were used for data analysis. Study 2 used survey data from 458 career-entry students to test the proposed moderated mediation model using PLS-SEM.

**Results:**

Artificial intelligence job replacement threat increased both challenge and hindrance appraisals. Challenge appraisal mediated the relationship between AI job replacement threat and career goal reengagement intention, whereas hindrance appraisal mediated the relationship between AI job replacement threat and career goal disengagement intention. Proactive personality strengthened the relationship between the AI job replacement threat and challenge appraisal, as well as the indirect effect on career goal reengagement intention. However, proactive personality did not significantly weaken the hindrance appraisal pathway.

**Discussion:**

The findings suggest that AI job replacement threat functions as a dual-meaning source of career uncertainty in the context of AI-driven labor market transformation. While proactive personality facilitates opportunity-oriented career adaptation, it does not eliminate perceptions of structural career threat, highlighting the limits of individual agency in navigating future work uncertainty.

## 1 Introduction

Artificial intelligence (AI) is rapidly reshaping the structure of the labor market, altering both job opportunities and the skills required for future employment (Yan-Ping and Ai-Qin, 2022; Aivaz et al., 2026). Against this backdrop, whether one’s future career may be replaced by AI has become an increasingly salient concern in university students’ career development. Unlike employees who have already entered organizational settings, university students are still at a critical stage of forming career goals, developing career identities, and preparing for labor market entry. At this stage, they do not yet hold stable occupational positions, but they need to respond to AI-driven job restructuring, skill renewal, and career uncertainty (Aivaz et al., 2026). Therefore, the AI job replacement threat is not merely a perceived employment risk associated with technological change. It may also serve as an important psychological stressor that shapes how university students adjust their career goals.

Currently, a growing body of research has investigated how AI and automation shape individual career development. Drawing on perspectives such as technology use, fairness, and subjective cognition, this literature has demonstrated how advances in AI technologies influence individuals’ psychological perceptions and behavioral responses (Robert et al., 2020; Cramarenco et al., 2023; Bakir et al., 2025). Nevertheless, despite these important advances, several limitations remain. First, prior research has predominantly focused on employees in organizational contexts, while giving comparatively limited attention to university students who are still at the career-entry stage. For university students, the AI job replacement threat not only implies uncertainty surrounding future employment opportunities but may also influence the formation, adjustment, and commitment of their career goals (Wong, 2024; Wu and Wen, 2025). Second, existing research has tended to conceptualize the AI job replacement threat as a unidimensional negative stressor and has primarily emphasized its adverse consequences. Less attention has been paid to why, when confronted with the same AI threat, different individuals may develop markedly different career goal adjustment responses (Bai et al., 2024; Wu and Wen, 2025; Zhao, 2025).

To address these limitations, this study draws on cognitive appraisal theory to develop a dual-appraisal model of how AI job replacement threat influences university students’ career goal adjustment intentions. Cognitive appraisal theory suggests that external stressors do not directly determine individuals’ psychological and behavioral responses. Rather, individuals’ subjective appraisals of a stressor are central to understanding their subsequent reactions (Lazarus and Folkman, 1984; Folkman et al., 1986). In the context of AI job replacement, university students may appraise AI-driven career uncertainty as a challenge, viewing it as a source of pressure that also creates opportunities to enhance their abilities, update their skills, and reshape their career paths. They may also appraise it as a hindrance, perceiving AI as reducing the controllability of personal effort and lowering the likelihood of attaining their original career goals (Folkman et al., 1986; Webster et al., 2011). Accordingly, this study argues that the AI job replacement threat may promote career goal reengagement intention (CGRI), that is, students’ intention to identify and invest effort in alternative meaningful career goals or pathways, through challenge appraisal, while increasing career goal disengagement intention (CGDI) through hindrance appraisal.

However, a focus on the mediating role of cognitive appraisal alone is not sufficient to fully explain the differentiated responses triggered by the AI job replacement threat. Existing research on the employment-related effects of AI has mainly examined the average effects of threat perception or its mediating mechanisms, while paying relatively limited attention to individual differences at the stage of appraisal formation (Bai et al., 2024; Zhao, 2025; Yuan and Liu, 2025). In other words, prior research has offered useful explanations of what consequences an AI threat may produce, but has provided less insight into why individuals may interpret the same threat as either a challenge or a hindrance. To address this theoretical gap, this study introduces proactive personality as a key boundary condition. Proactive personality refers to a stable tendency to identify opportunities, take action, and influence one’s environment (Seibert et al., 1999). Given that the AI job replacement threat is future-oriented, dynamically evolving, and to some extent open to coping efforts, individuals’ tendency to actively respond to environmental change may play an important role in their appraisal process. Proactive personality may therefore help explain why individuals form different cognitive appraisals when facing an AI job replacement threat, and how these appraisals further shape their career goal adjustment intentions.

Based on this reasoning, this study aims to explain how and when the AI job replacement threat influences university students’ career goal adjustment intentions. Specifically, it examines whether the AI job replacement threat affects career goal reengagement intention and CGDI through challenge appraisal and hindrance appraisal, respectively. It also investigates the role of proactive personality in the formation of cognitive appraisals. Through this research, we seek to show that the AI job replacement threat does not necessarily lead to a single negative outcome. Rather, its effects depend on individuals’ cognitive appraisal of the threat and their tendency to actively respond to changes in the career environment. Accordingly, this study makes three theoretical contributions. First, it extends research on AI job replacement threats to the context of university students’ career development, thereby addressing an important issue concerning the formation and adjustment of young people’s career goals in the AI era. Second, drawing on cognitive appraisal theory, it reveals the dual mechanisms through which the AI job replacement threat operates, moving beyond the tendency to view it solely as a negative stressor. Third, it introduces proactive personality as a boundary condition in the appraisal formation process, thereby explaining why individuals may respond differently to the same threat context.

## 2 Literature review and hypothesis development

### 2.1 AI job replacement threat

AI job replacement threat refers to individuals’ subjective perception that AI technologies may reduce their future career opportunities, replace jobs in their intended occupational fields, or weaken the market value of their existing knowledge and skills (Brougham and Haar, 2018; Wang and Wang, 2022). With the rapid diffusion of generative AI and algorithmic automation, this threat has become increasingly salient in recent employment and career research. Recent studies have shown that concerns about being replaced by AI are not limited to current employees, but also extend to individuals who are preparing for future labor-market entry (Jo and Park, 2025; Liang and Zhai, 2025). Unlike general job insecurity, which mainly refers to the possible loss or instability of one’s current job (Greenhalgh and Rosenblatt, 1984; Sverke et al., 2002), the AI job replacement threat is more anticipatory and future-oriented. Prior research has commonly defined job insecurity as individuals’ subjective perception that the continuity of their current job is under threat, emphasizing uncertainty around an “existing job” or a “current employment relationship.” For example, Greenhalgh and Rosenblatt (1984) define job insecurity as individuals’ powerlessness to maintain desired continuity in a threatened job situation, while Sverke et al. (2002) regard it as the subjective anticipation of involuntary job loss. For university students at the career-entry stage, this distinction is particularly important because they have not yet obtained stable organizational positions but have already begun to form career expectations, explore career paths, and prepare for future employment (Kosine and Lewis, 2008; Blokker et al., 2023). University is generally regarded as an important period for career exploration and career decision-making, during which individuals need to build a fit between self-understanding, career interests, and external career opportunities.

The AI job replacement threat also differs from broader AI anxiety or technology anxiety. AI anxiety mainly emphasizes the tension, fear, worry, or discomfort individuals experience when facing AI technologies (Wang and Wang, 2022). Its scope may include anxiety about learning AI, understanding how AI operates, interacting with AI, and how AI is configured. Job replacement is only one more specific career-related dimension of AI anxiety (Wang and Wang, 2022). Therefore, the AI job replacement threat is not merely a general fear of AI technology itself. Instead, it more specifically refers to the potential influence of AI on individuals’ career attainability, employment competitiveness, and the feasibility of their future career paths. For university students, this threat does not simply originate from the technology itself, but is closely related to whether their intended career paths remain viable in the context of AI-driven change. Recent evidence further suggests that students’ awareness of AI’s employment impact is positively associated with their perceived employment risk, indicating that AI-related career concerns have become an important part of students’ employment preparation and career decision-making (Liang and Zhai, 2025). In addition, research on university students has begun to show that AI-related anxiety may be interpreted through both challenge and hindrance appraisal processes, suggesting that AI job replacement concerns may involve both opportunity-oriented and threat-oriented meanings (Shi et al., 2026). Existing research also shows that AI job replacement anxiety influences students’ AI learning behavior and learning motivation, suggesting that students’ perceptions of AI replacement risk have already become linked to their preparation for future capabilities and their judgments about career development (Wang et al., 2024). These conceptual clarifications provide the basis for further examining how university students understand and respond to AI-related career uncertainty.

### 2.2 AI job replacement threat and career goal adjustment intention

Career goal adjustment intention refers to individuals’ psychological tendency to regulate their career goals when their original career goals become difficult to pursue or less viable because of changes in the career environment or obstacles to goal attainment (Wrosch et al., 2003). CGRI emphasizes individuals’ willingness to identify, commit to, and invest effort in alternative meaningful career goals or career pathways when their original career goals become difficult to pursue (Wrosch et al., 2003). CGDI, by contrast, refers to a tendency to withdraw effort and commitment from original career goals and to psychologically let go of these goals (Wrosch et al., 2003; Hu et al., 2017). Thus, career goal adjustment is not a single-directional response. Rather, it includes two relatively independent forms of adaptation: redirecting effort toward alternative meaningful career goals and withdrawing effort and commitment from original career goals.

In the context of AI job replacement, career goal adjustment intention should not be understood simply as a direct outcome of the AI job replacement threat. The AI job replacement threat is characterized by considerable ambiguity and duality. On the one hand, it may lead individuals to perceive a reduction in target-career positions, a decline in the value of their existing professional skills, and intensified employment competition (Wu and Wen, 2025; Zhao, 2025). On the other hand, it may also signal the emergence of new career opportunities, changes in the structure of skill demands, and the reshaping of career development paths (Voigt and Strauss, 2025; Zheng et al., 2025). This is especially the case for university students who have not yet formally entered the labor market. For them, AI-driven career uncertainty does not always point to losses that have already occurred. Instead, it more often involves a reassessment of future career possibilities (Voigt and Strauss, 2025; Ge et al., 2025). Therefore, when facing the same AI replacement threat, university students may develop different or even opposite career goal adjustment tendencies. Directly assuming a single linear relationship between AI job replacement threat and career goal adjustment intention would risk overlooking individuals’ subjective interpretation of the threat.

### 2.3 The challenge pathway: from AI job replacement threat to CGRI via challenge appraisal

Challenge appraisal refers to a cognitive judgment in which individuals interpret a stressful situation as involving potential for growth, learning, or achievement (Cavanaugh et al., 2000; Searle and Auton, 2015). According to cognitive appraisal theory, a stressor does not directly determine individual responses. Its effects depend on how individuals evaluate the relationship between the stressor and their own goals, resources, and coping capacity (Folkman et al., 1986; Webster et al., 2011). When individuals believe that a stressful situation is demanding but can still be managed through resource mobilization, capability development, or strategic adjustment, they are more likely to appraise it as a challenge. Such an appraisal may further stimulate active coping, task engagement, and adaptive behavior (LePine, 2022; Podsakoff et al., 2023; Liao et al., 2026).

In the context of AI job replacement, a higher AI job replacement threat means that individuals more strongly perceive technological change, skill renewal pressure, and shifts in career competition in the future occupational environment (Dinh, 2025). However, this threat does not necessarily represent only career loss. It may also signal the emergence of new career opportunities, changes in the structure of skill demands, and the reshaping of career paths. Prior research suggests that AI is changing how individuals work and how they view their long-term career prospects. It may create new opportunities by augmenting individual capabilities, improving work efficiency, and promoting innovation, but it may also generate a job replacement threat because it can perform tasks previously carried out by humans (Dinh, 2025; Voigt and Strauss, 2025). At the same time, when facing AI-related career shocks, individuals may need to reassess the suitability of their existing career goals and adapt to technological change by redirecting attention and effort toward adjusted or alternative career goals and pathways (Akkermans et al., 2021; Voigt and Strauss, 2025; Yuan and Liu, 2025). For university students at the career-entry stage, a high AI job replacement threat may make future labor market changes more salient. This may encourage them to interpret the situation as a developmental challenge that requires active response, renewal of capability structures, and stronger career preparation. Accordingly, we propose:

*H1*: In high (vs. low) AI job replacement threat conditions, students report higher challenge appraisal.

Furthermore, challenge appraisal may promote CGRI. Prior research suggests that when individuals believe a goal remains valuable and that feasible pathways for attaining it still exist, they are more likely to maintain goal commitment and reinvest resources (Wrosch et al., 2003; Hamm et al., 2022). Challenge appraisal strengthens individuals’ perception that a stressful situation is manageable and contains developmental opportunities, thereby facilitating active goal-regulation responses (Nicholls et al., 2016; Searle and Auton, 2015).

Thus, when university students appraise the AI job replacement threat as a challenge, they are more likely to believe that an AI-driven career change does not mean the end of meaningful career development. Instead, it may indicate the need to explore alternative meaningful career directions, adjust career pathways, and redirect effort toward new or revised career goals. In this case, the AI job replacement threat may be transformed into CGRI through challenge appraisal (Nicholls et al., 2016). Accordingly, we propose:

*H2*: Challenge appraisal mediates the relationship between AI job replacement threat and CGRI.

### 2.4 The hindrance pathway: from AI job replacement threat to CGDI via hindrance appraisal

Hindrance appraisal refers to a cognitive judgment in which individuals interpret a stressful situation as limiting goal attainment, weakening personal control, or obstructing future development (Cavanaugh et al., 2000; Searle and Auton, 2015). According to cognitive appraisal theory, when individuals perceive a stressor as beyond their control and as likely to impair goal attainment or resource acquisition, they are more likely to appraise it as a hindrance (Cavanaugh et al., 2000; Searle and Auton, 2015). Research on challenge–hindrance appraisal has also shown that hindrance appraisal is typically associated with negative emotions, avoidance-oriented coping, and lower levels of engagement, because individuals tend to interpret such situations as external constraints that are difficult to overcome (LePine, 2022; Podsakoff et al., 2023; Liao et al., 2026).

In the context of AI job replacement, a higher AI job replacement threat means that university students more strongly perceive the possibility that their intended future careers may be replaced by technology, that their professional skills may lose value, and that employment competition may intensify (Deng and Sun, 2026; Aivaz et al., 2026). Unlike general career stressors, the AI job replacement threat is marked by strong externality and uncontrollability: individuals can hardly directly change the pace of technological development, the employment structure of industries, or organizational decisions to adopt AI. For university students who have not yet entered a stable career track, high levels of AI job replacement threat may lead them to believe that the pathways for attaining their original career goals are restricted, thereby weakening their perceived control over future career development (Cheng et al., 2023; Hu et al., 2026). As a result, students are more likely to appraise the AI job replacement threat as a hindrance. Accordingly, we propose:

*H3*: In high (vs. low) AI job replacement threat conditions, students report higher hindrance appraisal.

In addition, hindrance appraisal may promote CGDI. When individuals appraise a stressor as limiting goal attainment, reducing personal control, and consuming resources without meaningful returns, continued investment in the original goal may appear less worthwhile (Podsakoff et al., 2023). In this case, disengagement can serve as a self-regulatory response to blocked goals, allowing individuals to reduce effort and commitment and avoid further resource loss (Brandstätter et al., 2013; Kappes and Schattke, 2022). Thus, students who appraise AI-related career change as a hindrance are more likely to develop an intention to disengage from their original career goals.

In the context of the AI job replacement threat, hindrance appraisal may explain how the perceived threat translates into CGDI. AI-related anxiety can heighten students’ concerns about unemployment risk and skill obsolescence, while hindrance appraisal in AI adoption contexts tends to elicit defensive and prevention-oriented responses (Cheng et al., 2023; Duan et al., 2026). When students perceive AI job replacement threats as making their target occupations less accessible and their skills less valuable, they may see continued investment in their original goals as less viable. Accordingly, the AI job replacement threat may increase CGDI through hindrance appraisal. Thus, we propose:

*H4*: Hindrance appraisal mediates the relationship between AI job replacement threat and CGDI.

### 2.5 The moderating role of proactive personality

Proactive personality refers to a stable tendency to identify opportunities, take action, and influence one’s surrounding environment (Seibert et al., 1999). Compared with general personality traits, proactive personality places greater emphasis on agency and future orientation when individuals face environmental change (Bateman and Crant, 1993; Seibert et al., 1999). Highly proactive individuals typically do not passively accept changes in the external environment. Instead, they are more likely to seek information, acquire resources, adjust strategies, and shape their future development paths (Seibert et al., 1999; Crant et al., 2016). Therefore, in the highly dynamic and uncertain career context of AI job replacement threat, a proactive personality may influence how university students cognitively appraise this threat.

Specifically, when the AI job replacement threat is high, students with a highly proactive personality are more likely to interpret it as a signal of a career change that requires an active response (Ohly and Fritz, 2010; Crant et al., 2016). They tend to focus on the skill renewal demands and career reshaping opportunities brought by AI development, and to believe that future labor market changes can be addressed through active learning, career exploration, and strategic adjustment (Cai et al., 2015; Yang and Jiang, 2025). Thus, proactive personality may strengthen the positive relationship between the AI job replacement threat and challenge appraisal. By contrast, students with a less proactive personality may be more inclined to passively accept external changes and less likely to seek coping resources or alternative pathways. As a result, they may be less likely to appraise the AI job replacement threat as a challenge.

At the same time, a proactive personality may also weaken the positive relationship between the AI job replacement threat and hindrance appraisal. Highly proactive individuals usually have a stronger tendency to shape their environment and take action. When facing an AI job replacement threat, they are therefore less likely to view it only as an uncontrollable external constraint, and more likely to search for room for intervention, adjustment, and coping (Crant et al., 2016; Nielsen et al., 2023). In other words, even when AI development makes the career environment more uncertain, highly proactive individuals are more likely to believe that such change is not entirely unchangeable, but can be adapted to through active learning, career exploration, and strategic adjustment (Wang et al., 2021; Yang and Jiang, 2025). Thus, even under high levels of AI job replacement threat, highly proactive students may be less likely to form strong hindrance appraisals. By contrast, less proactive students may be more likely to interpret an AI threat as an external pressure that weakens career controllability and goal attainability. Accordingly, we propose:

*H5a*: Proactive personality strengthens the relationship between the AI job replacement threat and challenge appraisal.

*H5b*: Proactive personality weakens the relationship between the AI job replacement threat and hindrance appraisal.

Furthermore, the moderating role of proactive personality in the formation of cognitive appraisals may also shape the indirect relationships between AI job replacement threat and career goal adjustment intentions. As argued above, challenge appraisal may promote CGRI, whereas hindrance appraisal may promote CGDI. If proactive personality strengthens the relationship between AI job replacement threat and challenge appraisal, the indirect effect of AI job replacement threat on CGRI through challenge appraisal should be stronger among students with a stronger proactive personality. In other words, highly proactive students may be more likely to transform the AI job replacement threat into an active process of career goal reengagement.

Correspondingly, if proactive personality weakens the relationship between AI job replacement threat and hindrance appraisal, the indirect effect of AI job replacement threat on CGDI through hindrance appraisal should be weaker among students with stronger proactive personality. That is, a proactive personality may reduce the likelihood that the AI job replacement threat is appraised as a hindrance and subsequently leads to career goal disengagement. Accordingly, we propose:

*H5c*: Proactive personality strengthens the indirect effect of AI job replacement threat on CGRI via challenge appraisal.

*H5d*: Proactive personality weakens the indirect effect of AI job replacement threat on CGDI via hindrance appraisal.

**Figure 1:**
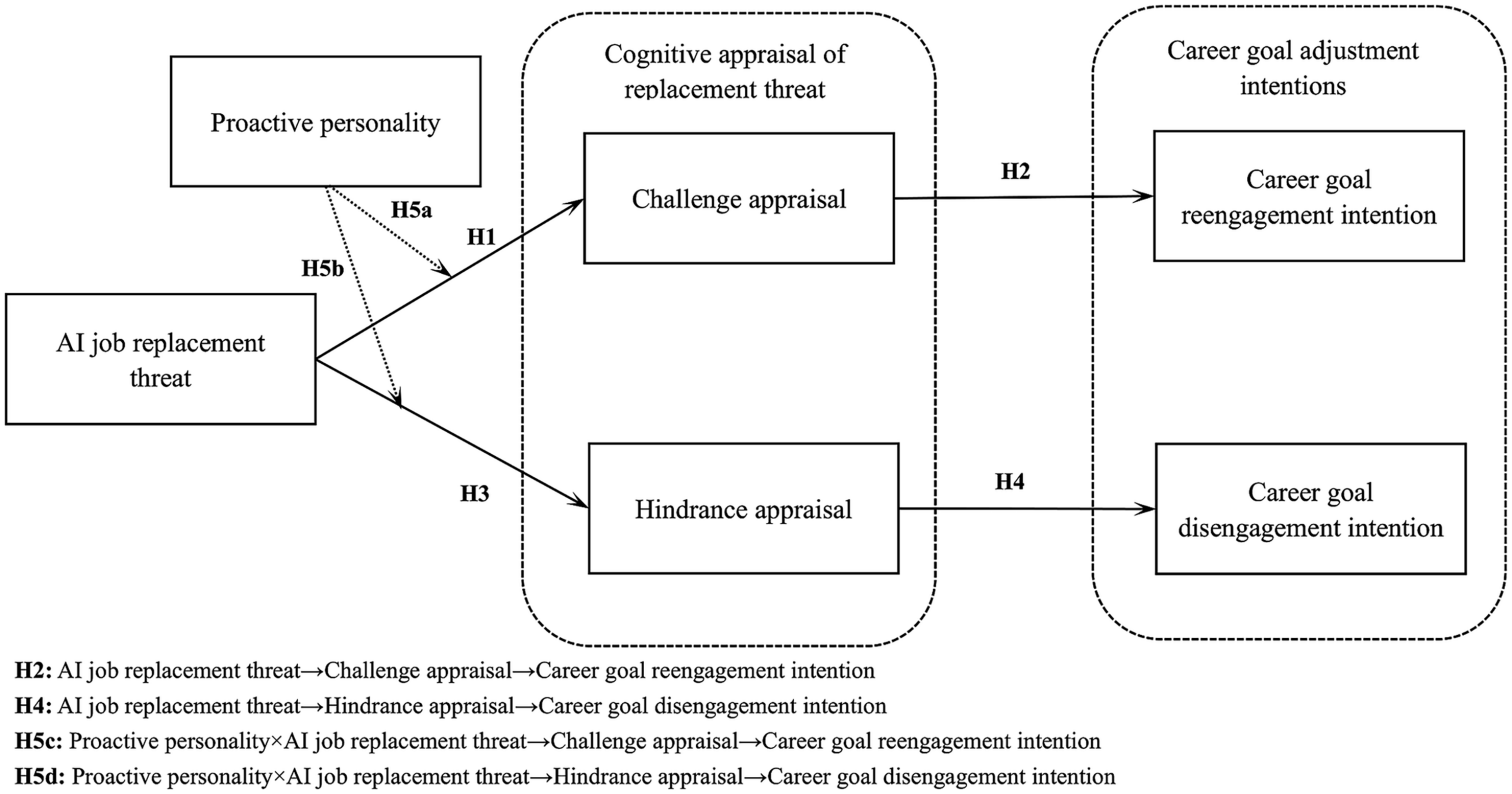
The conceptual model of this study.

Based on the above theoretical reasoning, this study develops an integrated model that places cognitive appraisal at the center of the mechanism. The model examines how the AI job replacement threat produces differentiated effects on university students’ career goal reengagement and disengagement intentions through challenge appraisal and hindrance appraisal. At the same time, proactive personality is introduced as a key boundary condition to explain individual differences in the formation of cognitive appraisals. The conceptual model of this study is presented in Figure 1.

## 3 Methodology

This study comprises two studies to examine the effects of AI job replacement threat on university students’ career goal adjustment intentions. Drawing on cognitive appraisal theory, we argue that AI job replacement threat influences students’ challenge and hindrance appraisals of future career development, which in turn affect their career goal reengagement and disengagement intentions. At the same time, a proactive personality may shape how individuals appraise the AI job replacement threat. The specific designs of the two sub-studies are presented in Table 1.

**Table 1:** Overview of research design.

Study	Research purpose	Participants	Main tests
Study 1	To examine the manipulation effect of the AI job replacement threat and test its mediating mechanism through cognitive appraisal	University students	Manipulation check; effects of AI job replacement threat on challenge and hindrance appraisals; mediating effects of cognitive appraisal on career goal reengagement and disengagement intentions
Study 2	To examine the mechanism of perceived AI job replacement threat in a real-world context and test the moderating role of proactive personality	Career-entry students	Effects of perceived AI job replacement threat on cognitive appraisal; mediating effects of challenge and hindrance appraisals; moderating effect of proactive personality; overall moderated mediation model

Study 1 adopts a scenario-based experimental design. By creating high and low AI job replacement threat conditions, it examines whether different levels of threat can induce different levels of perceived AI job replacement threat among university students. On this basis, Study 1 uses independent-samples t-tests to examine the effects of AI job replacement threat on challenge appraisal and hindrance appraisal. It then applies partial least squares structural equation modeling (PLS-SEM) to test the indirect effects of the AI job replacement threat condition on career goal reengagement and disengagement intentions through challenge and hindrance appraisals. This study is mainly designed to validate the scenario manipulation and provide initial evidence for the proposed mechanism.

Study 2 adopts a questionnaire survey design and collects data from university students preparing to enter the labor market. It further examines the mechanism through which perceived AI job replacement threat affects career goal adjustment intentions. Building on this design, Study 2 introduces proactive personality as a moderator and examines its moderating role in the relationship between perceived AI job replacement threat and cognitive appraisals. Given that the research model includes multiple mediation and moderation relationships, Study 2 also uses PLS-SEM for data analysis.

## 4 Study 1

### 4.1 Participants and procedure

Study 1 adopted a two-group between-subjects scenario-based experimental design, with 120 participants recruited for each group. Data were collected offline from a Chinese public full-time undergraduate institution in December 2025. The purpose of Study 1 was to examine the effectiveness of the AI job replacement threat manipulation and to provide an initial test of university students’ cognitive appraisals and career goal adjustment intentions under different threat conditions. Because this study focuses on university students who have not yet formally entered the labor market but are in the process of forming career goals and employment expectations, the offline experimental setting allowed the scenario materials to be presented in a relatively consistent response environment and helped reduce additional interference caused by differences in online response conditions.

The experiment was conducted using paper questionnaires or on-site questionnaires. Before completing the questionnaire, participants were informed that the study concerned university students’ views on AI technologies and future career development. They were also told that participation was voluntary, responses were anonymous, and the data would be used only for academic research. Participants then provided basic demographic information and reported the occupational field they intended to enter after graduation, so that they could relate the scenario materials to their own future career plans. After completing this information, participants were randomly assigned to either the high AI job replacement threat condition or the low AI job replacement threat condition. The two sets of materials were kept consistent in theme, structure, and length, with the main difference lying in the degree to which AI was described as threatening students’ future employment opportunities and intended occupational fields. After reading the scenario materials, participants completed measures of the manipulation check, challenge appraisal, hindrance appraisal, CGRI, and CGDI in sequence. All core variables were measured using 7-point Likert scales. To ensure data quality, an attention-check item was included in the questionnaire. Responses that failed the attention check, were incomplete, or showed obvious careless response patterns were excluded. The final valid sample consisted of 205 participants, including 108 in the high AI job replacement threat condition and 97 in the low AI job replacement threat condition. Among them, 48.8% were male, and 51.2% were female, with an average age of 20.87 years.

### 4.2 Measurement

All variables in Study 1 were adapted from established scales and contextualized to fit the career cognition and goal adjustment context in which university students face an AI job replacement threat. Except for demographic variables, all items were measured on a seven-point Likert scale. In adapting the items, the general work or goal contexts in the original scales were revised to refer to situations such as “AI may affect future employment opportunities” and “AI may replace some jobs in the target occupational field.” This ensured consistency between the measurement items and the scenario materials used in Study 1. Specifically, the AI job replacement threat items were adapted from Wang and Wang (2022), the challenge and hindrance appraisal items from Searle and Auton (2015), and the career goal reengagement and disengagement intention items from Wrosch et al. (2003).

The AI job replacement threat was used as the manipulation check. It measured the extent to which participants perceived, after reading the scenario materials, that AI might replace future employment opportunities and jobs in their target occupational fields. This variable included 6 items. A sample item is: “I am concerned that AI technologies may replace some jobs in the career field I intend to enter.” Challenge appraisal and hindrance appraisal were used to measure participants’ two types of cognitive appraisal of the AI job replacement situation. Challenge appraisal included 4 items. A sample item is: “I perceive that this situation will push me to explore new possibilities.” Hindrance appraisal also included 4 items. A sample item is: “I feel that this situation will restrict my ability to fully use my capabilities.” Career goal adjustment intention included CGRI and CGDI. CGRI included 6 items and measured whether participants tended to consider, identify, and invest effort in alternative meaningful career goals or career pathways when their original career goals became more difficult to attain because of AI replacement. A sample item is: “I would intend to seek other meaningful career directions.” CGDI included 4 items, two of which were reverse-coded. It measured whether participants tended to reduce their investment in their original career goals and let go of those goals. A sample item is: “I would intend to stop thinking about my original career goal and let it go.”

### 4.3 Manipulation check

To examine whether the scenario materials effectively distinguished between high and low AI job replacement threat, participants’ perceived AI job replacement threat after reading the materials was used as the manipulation-check variable. An independent-samples t-test was then conducted to compare the two groups. The results showed that participants in the high AI job replacement threat condition reported significantly higher perceived AI job replacement threat than those in the low AI job replacement threat condition [*M*high threat = 4.8935, *SD* = 0.94979; *M*low threat = 2.8969, *SD* = 0.82301; *t*(203) = 16.00, *p* < 0.001, Cohen’s *d* = 2.24]. This result indicates that the scenario materials effectively manipulated participants’ perceptions of AI job replacement threat and were therefore suitable for subsequent hypothesis testing.

### 4.4 Results

After confirming that the scenario materials successfully manipulated participants’ perceived AI job replacement threat, we further examined whether participants in the high and low AI job replacement threat conditions differed in their cognitive appraisals. In the subsequent analyses, the AI job replacement threat condition was coded as 0 = low AI job replacement threat condition and 1 = high AI job replacement threat condition. Table 2 presents the means, standard deviations, and correlations among the Study 1 variables.

**Table 2:** Descriptive statistics and correlations among variables (Study 1).

Variable	Mean	*SD*	1	2	3	4	5	6	7
1. Gender	0.490	0.501							
2. Age	20.87	1.427	0.049						
3. AI usage frequency	2.980	1.017	−0.063	−0.110					
4. AIJRT condition	0.530	0.501	−0.033	−0.012	0.054				
5. CA	3.794	1.471	0.001	0.049	0.038	0.524**			
6. HA	4.018	1.429	0.106	−0.011	−0.010	0.461**	0.409**		
7. CGRI	3.560	1.322	0.015	−0.016	0.046	0.167*	0.439**	0.018	
8. CGDI	4.095	1.314	0.022	0.102	−0.036	0.361**	0.303**	0.566**	0.024

Gender was coded as 1 = male and 0 = female, AIJRT condition was coded as 0 = low AI job replacement threat condition and 1 = high AI job replacement threat condition, AI usage frequency was measured on a 4-point scale, with higher scores indicating more frequent AI use, AIJRT AI job replacement threat, CA challenge appraisal, HA hindrance appraisal, CGRI career goal reengagement intention, CGDI career goal disengagement intention, *p < 0 0.05, **p < 0.01.

To examine whether the AI job replacement threat condition influenced participants’ cognitive appraisals, independent-samples t-tests were conducted for challenge appraisal and hindrance appraisal, respectively. The results showed that participants in the high AI job replacement threat condition reported significantly higher challenge appraisal than those in the low AI job replacement threat condition [*M*high threat = 4.523, *SD* = 1.266; *M*low threat = 2.982, *SD* = 1.244], *t*(203) = 8.774, *p* < 0.001, Cohen’s *d* = 1.23. Similarly, participants in the high-threat condition also reported significantly higher hindrance appraisal than those in the low-threat condition (*M*high threat = 4.641, *SD* = 1.229; *M*low threat = 3.325, *SD* = 1.316), *t*(203) = 7.406, *p* < 0.001, Cohen’s *d* = 1.04. These findings indicate that the high AI job replacement threat condition elicited both stronger challenge and hindrance appraisals, thereby supporting H1 and H3.

The indirect effects were then examined using PLS-SEM with bootstrapped confidence intervals. The result shows that the indirect effect of the AI job replacement threat condition on CGRI through challenge appraisal was significant, *β* = 0.465, *t* = 6.095, *p* < 0.001, 95% *CI* [0.326, 0.626]. The indirect effect of AI job replacement threat condition on CGDI through hindrance appraisal was also significant, *β* = 0.527, *t* = 5.992, *p* < 0.001, 95% *CI* [0.371, 0.714]. H2 and H4 were supported. Thus, Study 1 provides initial experimental evidence for the dual-appraisal mechanism. High AI job replacement threat increased both challenge and hindrance appraisals, which are further related to different forms of career goal adjustment intention.

## 5 Study 2

### 5.1 Participants and procedure

Study 2 adopted a cross-sectional questionnaire survey design. The participants were career-entry students currently enrolled in junior college, undergraduate, or postgraduate programs in China. Because this study focuses on the effect of AI job replacement threat on career goal adjustment intentions, the sample was primarily limited to students engaged in career planning, internship preparation, or job search preparation. This group is at a critical stage of transition from school to the labor market and typically needs to evaluate future career opportunities, the fit between professional skills and job requirements, and changes in the employment environment. It is therefore well aligned with the context of this study. Given the focus on students’ career-entry status and their subjective perceptions of AI job replacement threat, the sample was not further divided by institutional type, such as public/government, private, or foreign universities. It should be noted that the present study did not aim to compare the objective level of AI replacement risk across academic disciplines. Rather, it focused on students’ subjective perception of AI job replacement threat and examined how such perceived threat was associated with different cognitive appraisals and career goal adjustment intentions.

Participants were recruited through university course channels, the university admissions and career services office, and social media groups commonly used by students. To improve sample relevance and data quality, screening questions were included at the beginning of the questionnaire. Only respondents who met the following criteria were retained: first, they were currently enrolled in a junior college, undergraduate, or postgraduate program; second, they were engaged in career planning, internship preparation, or job-search preparation; and third, they were able to understand the descriptions of AI technology and future career development in the questionnaire. Responses that did not meet these criteria were excluded from subsequent analyses.

The questionnaire included measures of the core variables, a marker variable, and demographic information. To reduce the potential influence of common method bias (CMB), several procedural controls were adopted in the questionnaire design. First, anonymity and confidentiality were emphasized at the beginning of the questionnaire to reduce evaluation concerns and social desirability bias. Second, relatively independent instructions and response contexts were provided for different constructs. Daily behavioral tendencies, AI job replacement threat evaluation, cognitive appraisals in the AI replacement context, and career goal adjustment intentions were presented separately, so as to reduce respondents’ direct inference of relationships among variables and their tendency toward consistency in responding. Finally, an attention-check item was embedded in the questionnaire. During data cleaning, responses that failed the attention check, had abnormal completion times, or showed obvious careless response patterns were removed. A total of 507 questionnaires were collected. According to the preset data-cleaning criteria, responses that failed to meet the screening conditions, failed the attention check, showed abnormal completion times, or were otherwise invalid were excluded. The final valid sample consisted of 458 participants. Among them, 47.2% were male, and 52.8% were female, with a mean age of 22.52 years. In terms of educational stage, 41.0% were junior college students, 40.8% were undergraduate students, and 18.1% were postgraduate students.

### 5.2 Measurement

Study 2 employed the primary measurement instruments from Study 1 to ensure consistency in assessing the core constructs across the two studies. Specifically, the items for AI job replacement threat, challenge appraisal, hindrance appraisal, CGRI, and CGDI were consistent with those used in Study 1. The difference is that Study 1 manipulated AI job replacement threat through scenario materials, whereas Study 2 directly measured participants’ subjective perceptions of AI job replacement threat in a real-world career planning context. Therefore, the relevant items in Study 2 were framed around the idea that “AI technologies may affect future employment opportunities and target occupational fields.” Except for demographic variables and AI usage frequency, all items were measured using 7-point Likert scales.

In addition to these core variables, Study 2 measured proactive personality to examine its moderating role in the relationship between AI job replacement threat and cognitive appraisal. Proactive personality was measured using the 10-item scale developed by Seibert et al. (1999). Sample items include “If I see something I don’t like, I fix it.” and “I excel at identifying opportunities.” To further assess potential CMB, Study 2 also included color preference as a marker variable. This variable consisted of four items measuring participants’ general color preferences in daily life, which were theoretically unrelated to the core variables of this study (Rönkkö and Ylitalo, 2011; Leonard et al., 2024). Finally, because individual background characteristics and prior exposure to AI may influence students’ judgments of AI-related career risks, gender, age, and AI usage frequency were controlled for in the subsequent analyses.

### 5.3 Non-response bias

To examine whether the sample was affected by non-response bias, this study used the early–late response comparison method. Based on the order in which questionnaires were returned, the first 25% of responses were classified as the early-response group, and the last 25% were classified as the late-response group. Independent-samples *t*-tests were then conducted on the main study variables. The results showed no significant differences between the two groups for any of the variables (*p* > 0.05). Therefore, the sample was considered unlikely to be affected by significant non-response bias.

### 5.4 CMB test

Because Harman’s single-factor test has limited ability to detect CMB and may underestimate potential method effects (Podsakoff et al., 2003), this study further adopted two more rigorous approaches: the common method factor method and the marker variable method. The results of the common method factor analysis showed that the average substantive factor loading of the items was 0.819, with an average explained variance of 0.673. In contrast, the average method factor loading was close to zero, and the average explained variance was only 0.003. These results indicate that item variance was mainly explained by the theoretical constructs rather than by common method factors (Liang et al., 2007).

In addition, this study used the marker variable method to further examine the influence of CMB on the main research relationships (Chin et al., 2013). After adding the marker variable, changes in the core path coefficients were very small. For example, the coefficient for AIJRT → CA changed only from 0.522 to 0.520, while the coefficient for AIJRT → HA remained 0.528. Moreover, none of the marker variable paths was significant. The changes in the explanatory power of the dependent variables were also minimal, with the largest ΔR^2^ being only 0.006. Therefore, the results of both the common method factor method and the marker variable method suggest that CMB did not seriously affect the findings of this study.

### 5.5 Evaluating the measurement model

This study first evaluated the measurement model in terms of reliability and convergent validity. The results showed that the item loadings for each construct were generally high, with most loadings exceeding 0.70 and falling within an acceptable range. In addition, Cronbach’s *α* ranged from 0.833 to 0.946, and composite reliability (CR) ranged from 0.889 to 0.953, both exceeding the recommended threshold of 0.70. These results indicate good internal consistency across the scales. Meanwhile, the average variance extracted (AVE) values ranged from 0.650 to 0.709, all above the threshold of 0.50, suggesting that the measurement items adequately explained their corresponding latent constructs and that convergent validity was satisfactory (Hair et al., 2019). Overall, all constructs demonstrated acceptable reliability and convergent validity.

This study further assessed discriminant validity. As shown in Table 3, the means and standard deviations of the constructs were within reasonable ranges, and the skewness and kurtosis values did not indicate serious distributional abnormalities. In terms of discriminant validity, all HTMT coefficients between constructs were below 0.85. In addition, the HTMT inference results showed that none of the confidence intervals included 1, indicating sufficient distinctiveness among the constructs (Henseler et al., 2015). Therefore, the measurement model met acceptable standards for internal consistency, convergent validity, and discriminant validity, and was suitable for subsequent structural model analysis.

**Table 3:** Descriptive statistics and discriminant validity (Study 2).

Constructs	Mean	S. D.	Skewness	Kurtosis	1	2	3	4	5	6
1. AIJRT	4.024	1.332	0.040	−1.115	**0.814**	0.579	0.325	0.237	0.600	0.080
2. CA	3.771	1.504	0.041	−1.261	0.511	**0.842**	0.256	0.577	0.262	0.083
3. CGDI	4.122	1.340	−0.263	−0.923	0.282	0.217	**0.817**	0.068	0.658	0.083
4. CGRI	3.556	1.324	0.345	−0.901	0.214	0.511	0.056	**0.806**	0.040	0.114
5. HA	4.044	1.437	−0.087	−1.169	0.528	0.225	0.554	0.012	**0.831**	0.044
6. PP	3.638	1.409	0.050	−1.498	−0.074	0.085	−0.047	0.116	−0.037	**0.818**

Diagonal = $\sqrt{\mathrm{AVE}\;}$ ; Below diagonal: estimated correlations; Above diagonal: HTMT coefficient of correlations, PP Proactive personality, AIJRT AI job replacement threat, CA Challenge appraisal, HA Hindrance appraisal, CGRI Career goal reengagement intention, CGDI Career goal disengagement intention. Bold diagonal values represent the square roots of the AVE for the corresponding constructs.

### 5.6 Hypothesis testing

This study tested the hypotheses using PLS-SEM. Path coefficients and significance levels were estimated through 5,000 bootstrap resamples. The moderation effects were examined by directly constructing the interaction term between proactive personality and AI job replacement threat. All analyses controlled for gender, age, and AI usage frequency. Regarding the structural model assessment, the *R*^2^ values of the endogenous variables ranged from 0.263 to 0.310, suggesting an acceptable level of explanatory power. The *Q*^2^ values ranged from 0.166 to 0.215 and were all greater than zero, indicating predictive relevance. In addition, the inner-model VIF values ranged from 1.000 to 1.006, which were below commonly used thresholds, suggesting that multicollinearity was unlikely to substantially bias the structural estimates.

The hypothesis testing results are shown in Table 4. AI job replacement threat was positively associated with challenge appraisal (*β* = 0.522, *p* < 0.001) and hindrance appraisal (*β* = 0.528, *p* < 0.001), supporting H1 and H3. The indirect association between AI job replacement threat and CGRI through challenge appraisal was significant (*β* = 0.267, *p* < 0.001), supporting H2. Similarly, the indirect association between AI job replacement threat and CGDI through hindrance appraisal was significant (*β* = 0.292, *p* < 0.001), supporting H4. Regarding the moderation effects, the interaction between proactive personality and AI job replacement threat was positively associated with challenge appraisal (*β* = 0.187, *p* < 0.001), supporting H5a. However, the interaction between proactive personality and AI job replacement threat was not significantly associated with hindrance appraisal (*β* = −0.032, *p* = 0.410), indicating that H5b was not supported. Further moderated mediation results showed that proactive personality significantly moderated the indirect association between AI job replacement threat and CGRI via challenge appraisal (*β* = 0.096, *p* < 0.001), supporting H5c. In contrast, proactive personality did not significantly moderate the indirect association between AI job replacement threat and CGDI via hindrance appraisal (*β* = −0.018, *p* = 0.412), indicating that H5d was not supported.

**Table 4:** Results of hypothesis testing (Study 2).

Paths	*β*-value	LLCI	ULCI	*p*-value	*R*^2^	*Q*^2^	Significance
H1: AIJRT → CA	0.522	0.442	0.594	< 0.001	0.310	0.215	Yes
H2: AIJRT → CA → CGRI	0.267	0.216	0.319	< 0.001			Yes
H3: AIJRT → HA	0.528	0.457	0.598	< 0.001	0.280	0.189	Yes
H4: AIJRT → HA → CGDI	0.292	0.239	0.351	< 0.001			Yes
H5a: PP x AIJRT → CA	0.187	0.099	0.26	< 0.001			Yes
H5b: PP x AIJRT → HA	−0.032	−0.111	0.044	0.410			No
H5c: PP x AIJRT → CA → CGRI	0.096	0.05	0.137	< 0.001	0.263	0.166	Yes
H5d: PP x AIJRT → HA → CGDI	−0.018	−0.061	0.025	0.412	0.309	0.200	No

LLCI, lower-limit of confidence interval; ULCI, upper-limit of confidence interval; PP, proactive personality; AIJRT, AI job replacement threat; CA, challenge appraisal; HA, hindrance appraisal; CGRI, career goal reengagement intention; CGDI, career goal disengagement intention.

### 5.7 Predictive assessment using PLSpredict

In addition to explanatory power, this study further assessed the out-of-sample predictive power of the model using PLSpredict (Hair et al., 2019; Shmueli et al., 2019). As shown in Table 5, all *Q*^2^predict values were above zero, indicating predictive relevance of the endogenous constructs. Furthermore, the majority of the prediction errors generated by the PLS-SEM model were lower than those of the linear model benchmark (LM), as reflected by the negative *Δ*RMSE and *Δ*MAE values across most indicators. Although two indicators (CGRI4 and CGRI5) showed slightly inferior prediction performance relative to the LM benchmark, the differences were marginal. Thus, the results suggest that the proposed model demonstrates moderate predictive power (Shmueli et al., 2019).

**Table 5:** PLSpredict results.

Indicator	*Q*^2^predict	RMSE (PLS-SEM)	RMSE (LM)	*Δ*RMSE	MAE (PLS-SEM)	MAE (LM)	*Δ*MAE
CA1	0.201	1.509	1.555	−0.046	1.222	1.263	−0.041
CA2	0.234	1.643	1.695	−0.052	1.362	1.391	−0.029
CA3	0.209	1.633	1.657	−0.024	1.337	1.358	−0.021
CA4	0.198	1.577	1.610	−0.033	1.301	1.319	−0.018
CGDI1	0.044	1.535	1.580	−0.045	1.244	1.277	−0.033
CGDI2	0.053	1.575	1.608	−0.033	1.275	1.303	−0.028
CGDI3	0.058	1.620	1.648	−0.028	1.350	1.364	−0.014
CGDI4	0.046	1.677	1.721	−0.044	1.389	1.418	−0.029
CGRI1	0.061	1.653	1.700	−0.047	1.349	1.387	−0.038
CGRI2	0.044	1.707	1.750	−0.043	1.428	1.457	−0.029
CGRI3	0.020	1.514	1.537	−0.023	1.265	1.283	−0.018
CGRI4	0.047	1.826	1.817	0.009	1.497	1.499	−0.002
CGRI5	0.040	1.539	1.536	0.003	1.277	1.254	0.023
CGRI6	0.023	1.408	1.425	−0.017	1.124	1.147	−0.023
HA1	0.125	1.616	1.629	−0.013	1.330	1.356	−0.026
HA2	0.226	1.496	1.528	−0.032	1.186	1.215	−0.029
HA3	0.202	1.502	1.518	−0.016	1.221	1.231	−0.010
HA4	0.189	1.633	1.654	−0.021	1.367	1.386	−0.019

CA, challenge appraisal; HA, hindrance appraisal; CGRI, career goal reengagement intention; CGDI, career goal disengagement intention.

## 6 Discussion

### 6.1 Discussion of results

This study examined the relationship between AI job replacement threat and university students’ career goal adjustment intentions across two sub-studies. To provide an overview of the study objectives, hypotheses, major findings, and implications, Table 6 summarizes the main results of the two studies.

**Table 6:** Summary of study objectives, hypotheses, findings, and implications.

Study objective	Hypotheses	Findings	Implications
To examine whether AI job replacement threat evokes different cognitive appraisals.	H1, H3	AI job replacement threat was positively associated with both challenge appraisal and hindrance appraisal.	AI job replacement threat has a dual meaning for career-entry students and should not be viewed only as a negative stressor.
To test whether cognitive appraisals explain career goal adjustment intentions.	H2, H4	Challenge appraisal mediated the relationship between AI job replacement threat and career goal reengagement intention, whereas hindrance appraisal mediated the relationship with career goal disengagement intention.	Different appraisal pathways explain why the same AI-related career uncertainty may lead to different forms of career goal adjustment.
To examine whether proactive personality shapes the appraisal process.	H5a, H5b	Proactive personality strengthened the relationship between AI job replacement threat and challenge appraisal, but did not significantly weaken the relationship with hindrance appraisal.	Proactive personality mainly enhances opportunity-oriented appraisal, but may not fully reduce perceptions of structural career threat.
To test the moderated mediation effects of proactive personality.	H5c, H5d	Proactive personality strengthened the indirect effect on career goal reengagement intention through challenge appraisal, but did not significantly weaken the indirect effect on career goal disengagement intention through hindrance appraisal.	The role of proactive personality is asymmetric: it facilitates adaptive reengagement but does not necessarily buffer disengagement-oriented responses.

Overall, the findings support the dual cognitive appraisal pathways proposed in this study. The scenario-based experiment in Study 1 showed that, compared with students in the low AI job replacement threat condition, those in the high-threat condition reported higher levels of both challenge appraisal and hindrance appraisal. The survey data in Study 2 further showed that perceived AI job replacement threat was positively associated with both types of cognitive appraisal. These findings suggest that, in the context of university students’ career planning, the AI job replacement threat is not associated with only a single negative appraisal. When students perceive that AI may affect their future employment opportunities and intended occupational fields, they may interpret it either as a signal of change that requires learning, adaptation, and career replanning or as an external constraint that weakens the attainability of their career goals. This finding is consistent with cognitive appraisal theory, which argues that individuals’ responses to a stressor are not determined solely by the stressor itself but depend on how they evaluate its relationship with their own goals, resources, and controllability (Lazarus and Folkman, 1984; Folkman et al., 1986). It also echoes research on challenge–hindrance appraisal, which suggests that the same stressful situation may contain both challenge and hindrance meanings (Webster et al., 2011; Searle and Auton, 2015).

The findings further show that the two types of cognitive appraisal are linked to different career goal adjustment intentions. Both Study 1 and Study 2 showed that challenge appraisal significantly mediated the relationship between AI job replacement threat and CGRI, whereas hindrance appraisal significantly mediated the relationship between AI job replacement threat and CGDI. Specifically, when students were more likely to appraise AI job replacement threat as a challenge involving opportunities for learning, adjustment, and coping, they reported higher levels of CGRI. By contrast, when students were more likely to appraise it as a hindrance that limits goal attainment or weakens career controllability, they reported higher levels of CGDI. This result is consistent with the basic logic of goal adjustment research. When individuals believe that a goal can still be adjusted or achieved through alternative pathways, reengagement may become an adaptive response. However, when individuals believe that the likelihood of goal attainment has declined and that continued investment is costly, disengagement may also serve as a self-regulatory way to reduce further resource loss (Wrosch et al., 2003; Brandstätter et al., 2013; Kappes and Schattke, 2022). Nevertheless, this study measured career goal adjustment intentions rather than subsequent career behaviors. Therefore, these findings should be understood as students’ psychological tendencies under AI threat, rather than as direct evidence of their actual career choices.

The results regarding proactive personality show an asymmetric pattern. Study 2 found that proactive personality significantly strengthened the relationship between AI job replacement threat and challenge appraisal. It also strengthened the indirect relationship between the AI job replacement threat and CGRI through challenge appraisal. This suggests that, in the present sample, students with higher proactive personality were more likely to identify room for learning, adjustment, and replanning when facing AI job replacement threat, and therefore formed stronger challenge appraisals. This finding is consistent with the proactive personality literature, which suggests that highly proactive individuals are generally more inclined to identify opportunities, acquire resources, and adjust their actions in response to environmental change (Seibert et al., 1999; Crant et al., 2016). However, proactive personality did not significantly weaken the relationship between AI job replacement threat and hindrance appraisal, nor did it weaken the indirect relationship between AI job replacement threat and CGDI through hindrance appraisal. Thus, the findings do not support the view that proactive personality generally buffers negative appraisals of AI threat. A more cautious interpretation is that proactive personality may mainly enhance individuals’ ability to identify actionable space within AI threat, but does not necessarily reduce their perceptions of job replacement, skill devaluation, and career uncertainty. Because AI job replacement threat involves broader external factors such as technological development and labor market change, even highly proactive students may still perceive realistic constraints on some career goals.

### 6.2 Theoretical implications

This study first refines the theoretical nature of the AI job replacement threat. Its psychological meaning is not fixed by the threat itself, but is constructed through individuals’ appraisal processes. The findings show that the AI job replacement threat is associated with both challenge appraisal and hindrance appraisal, suggesting that it does not carry only a single negative meaning in the context of career goal adjustment. For university students, the perception that AI may replace future jobs or weaken the value of their skills may indicate constraints on their career paths. At the same time, it may also signal the need to reconsider career goals, capability structures, and future development paths. In this sense, this study further specifies the explanatory role of cognitive appraisal theory in the context of AI-related career development: the effect of AI job replacement threat is not directly determined by the level of threat itself, but is given different psychological meanings through the two pathways of challenge appraisal and hindrance appraisal. This theoretical treatment moves the AI job replacement threat beyond a risk variable that merely points to anxiety or insecurity, and instead conceptualizes it as a career-context signal with appraisal openness (Folkman et al., 1986; Webster et al., 2011; Searle and Auton, 2015).

Building on this argument, this study further extends the logic of dual appraisal to the process of career goal adjustment. It shows that the AI job replacement threat does not connect with all career responses in the same way. The findings indicate that challenge appraisal is associated with CGRI, whereas hindrance appraisal is associated with CGDI. This finding clarifies the differentiated formation logic of career goal reengagement and career goal disengagement. The former is based more on individuals’ judgments about learning opportunities, the possibility of pathway adjustment, and future coping capacity. The latter is based more on their judgments that goals are constrained, controllability is reduced, and continued investment has lower value. Therefore, this study does not simply link the AI threat to career goal adjustment intention. Instead, it reveals how different appraisal pathways lead to different types of goal-regulation intentions. This approach helps avoid treating reengagement simply as positive persistence and disengagement simply as negative withdrawal. Rather, it places both responses within different appraisal logics and understands them as differentiated regulatory strategies for dealing with career uncertainty (Wrosch et al., 2003; Brandstätter et al., 2013).

Furthermore, this study not only reveals how the AI job replacement threat affects career goal adjustment intentions through cognitive appraisal, but also explains why this appraisal process differs across individuals. The results for proactive personality show that it significantly strengthens the relationship between AI job replacement threat and challenge appraisal, as well as the indirect relationship leading to CGRI through challenge appraisal. However, it does not significantly weaken the relationship between the AI job replacement threat and hindrance appraisal, nor the indirect relationship leading to CGDI through hindrance appraisal. This asymmetric pattern deepens the theoretical explanation. In the context of the AI job replacement threat, proactive personality does not appear to reduce threat perception or hindrance appraisal in a general way. Instead, it more specifically enhances individuals’ recognition of actionable space, learning possibilities, and opportunities for pathway reconstruction. In other words, in a context of technological change characterized by externality and structural constraints, individual proactivity may be better at amplifying challenge-based interpretations than at offsetting hindrance-based interpretations. In this way, the study offers a more specific account of the functional boundary of proactive personality and advances the “dual appraisal–career goal adjustment intention” model from a general mechanism to a conditional mechanism involving individual differences (Seibert et al., 1999; Crant et al., 2016).

### 6.3 Practical implications

This study suggests that university students should avoid interpreting the AI job replacement threat simply as a signal that their career prospects are damaged or that their original career goals are no longer feasible. For students who are engaged in career planning, internship preparation, or job-search preparation, AI-related uncertainty is often reduced to a broad question of whether future jobs will be replaced. However, such a general judgment is difficult to translate into effective action. A more practical approach is to break down the AI threat into specific career assessment questions: which tasks in the target occupation are more likely to be replaced by AI, which abilities still retain strong human advantages, how existing professional knowledge can be combined with AI tools, data-related capabilities, or interdisciplinary skills, and whether adjacent career paths are available. Through such concrete assessment, students can avoid giving up their original career goals too early because of excessive anxiety, while also avoiding blind optimism that overlooks real pressure for skill renewal. Career preparation in the AI era should therefore move beyond a binary choice between persistence and abandonment, and instead focus on continuous goal adjustment based on career information, skill gaps, and personal resources.

Beyond students’ own need to strengthen career assessment capacity, university career services and employment guidance courses should also move beyond simple information provision and place greater emphasis on developing students’ cognitive appraisal and goal-regulation abilities. Traditional career guidance often focuses on résumé revision, interview training, job information delivery, and employment policy explanation. Yet, as AI rapidly changes occupational structures, providing employment information alone is not sufficient to help students understand the specific implications of technological change for their career goals. Universities may incorporate AI career impact assessment modules into career planning courses, guiding students to analyze task changes, job restructuring, and skill transfer pathways in their target industries. They may also use case discussions, career scenario simulations, and reflective writing to help students distinguish which AI-related changes can be addressed as challenges through learning and pathway adjustment, and which changes require a reassessment of the realistic constraints on their original goals. At the same time, career counseling should not merely encourage students to maintain a positive attitude in general terms. Instead, it should help them develop more realistic goal adjustment strategies: for career goals that still have feasible pathways, students should be supported in skill supplementation and pathway redesign; for goals whose feasibility has clearly declined, students should be helped to identify alternative goals to reduce ineffective investment and persistent frustration.

Furthermore, efforts at the student and university levels need to be supported by clearer external career signals. Educational administrators, industry organizations, and employers can reduce the likelihood that students form excessive threat perceptions or engage in blind reengagement under conditions of insufficient information by communicating career-related information more transparently. Universities can collaborate with firms, industry associations, and alumni networks to regularly update students on AI applications, job changes, and capability requirements across different occupational fields, enabling students to plan their careers based on more accurate judgments of the external environment. In campus recruitment, internship programs, and career talks, employers can also explain more clearly how AI tools are actually used in specific jobs, how job tasks are changing, and what capabilities are required, rather than simply emphasizing “AI capability” or “digital literacy” in broad terms. In addition, for students with lower proactive personality, weaker access to career information, or a stronger tendency to interpret AI-related change as an uncontrollable hindrance, universities and employers can provide more structured support, such as AI skills training, career exploration programs, mentoring, and low-risk internship experiences. Such support can help students translate the AI job replacement threat into more concrete action plans, rather than remaining in abstract anxiety or premature disengagement.

### 6.4 Limitations and future directions

Although this study combined a scenario-based experiment and a questionnaire survey to examine the relationship between AI job replacement threat and university students’ career goal adjustment intentions, several limitations remain. In Study 1, the experimental scenario helped control the level of AI job replacement threat, but the scenario materials were still a simplified representation of the real career environment. Study 2 collected data in a real-world career planning context, but its cross-sectional and survey-based design limits further conclusions about dynamic changes among variables, causal directions, and the detailed meanings students attach to AI-related career uncertainty. Future research could therefore adopt longitudinal, multi-wave, or mixed-method designs, including interviews or open-ended responses analyzed with qualitative software such as NVivo, to examine how AI job replacement threat, cognitive appraisals, and career goal adjustment intentions change over time and how students make sense of AI-related career uncertainty as they move through career planning, internships, job search, and the transition from university to work. Such designs could also test whether these intentions are translated into actual career choices.

Second, this study focused on career-entry students engaged in career planning, internship preparation, or job-search preparation. This sample is appropriate for examining AI-related career uncertainty before labor market entry, but it may limit the generalizability of the findings to other career stages. Future research could extend the sample to interns, recent graduates, or early-career employees to test the model across different career development stages. Moreover, career-entry students are not homogeneous. Their academic disciplines and intended occupational fields may shape how they understand AI-related career change. Students in technology-oriented fields may view AI as creating opportunities for skill development and career advancement, whereas students in other fields may perceive stronger risks of job displacement or skill devaluation. Such differences may affect perceived AI job replacement threat and the relative strength of challenge and hindrance appraisals. Future research could compare students from different disciplinary backgrounds or examine whether academic discipline moderates the proposed appraisal pathways.

Finally, this study focused mainly on the roles of challenge appraisal, hindrance appraisal, and proactive personality. However, the effects of the AI job replacement threat on career goal adjustment may also be shaped by other individual and contextual resources. For example, AI self-efficacy, career adaptability, career control, social support, and the quality of university career services may all influence how students understand and respond to the AI replacement threat. At the same time, this study measured career goal adjustment intentions rather than actual career behaviors. Future research could incorporate behavioral or tracking data, such as course choices, participation in skills training, internship position selection, and changes in job-search direction, to more directly examine how the AI job replacement threat affects students’ real career development trajectories.

## Data Availability

The supplementary analytical results supporting the conclusions of this article are included in the article and its Supplementary material. The anonymized raw data supporting the findings of this study are available from the corresponding author upon reasonable request.
