# Supplementary material for: When AI threatens future careers: a dual-appraisal model of university students’ career goal adjustment intentions
Source: Front Psychol. 2026 Jul 8;17:1879844. doi: 10.3389/fpsyg.2026.1879844 (PMC13388150; doi:10.3389/fpsyg.2026.1879844)
Supplement: Supplementary file 1 [file Table_1.DOCX]

Supplementary Material

**Appendix 1.** Independent-samples *t*-test results for cognitive appraisals(Study 1)

| Variable | Low threat *M*(*SD*) | High threat *M*(*SD*) | *t*-value | *p*-value | Cohen’s *d* |
| --- | --- | --- | --- | --- | --- |
| CA | 2.982 (1.244) | 4.523 (1.266) | 8.774 | <0.001 | 1.23 |
| HA | 3.325 (1.316) | 4.641 (1.229) | 7.406 | <0.001 | 1.04 |

Low threat condition: n = 97, high threat condition: n = 108, Cohen’s *d* was calculated based on pooled standard deviations, *p*-values are two-tailed, *CA* Challenge appraisal, *HA* Hindrance appraisal.

**Appendix 2.** Mediation analysis results(Study 1).

| Paths | *β*-value | *LLCI* | *ULCI* | *p*-value | Significance |
| --- | --- | --- | --- | --- | --- |
| AIJRT → CA→ CGRI | 0.465 | 0.326 | 0.626 | <0.001 | Yes |
| AIJRT → HA → CGDI | 0.527 | 0.371 | 0.714 | <0.001 | Yes |

AIJRT condition was coded as 0 = low AI job replacement threat condition and 1 = high AI job replacement threat condition, *LLCI* Lower-limit of confidence interval, *ULCI* Upper-limit of confidence interval, *AIJRT* AI job replacement threat, *CA* Challenge appraisal, *HA* Hindrance appraisal, *CGRI* Career goal reengagement intention, *CGDI* Career goal disengagement intention.

**Appendix 3.** Comparison between early and late respondents(Study 2).

| Constructs | Early (Mean) | Late (Mean) | *t*-value | *p*-value |
| --- | --- | --- | --- | --- |
| PP | 3.643 | 3.530 | 0.605 | 0.546 |
| AIJRT | 3.903 | 4.164 | -1.467 | 0.144 |
| CA | 3.659 | 3.724 | -0.326 | 0.745 |
| HA | 3.974 | 4.057 | -0.432 | 0.666 |
| CGRI | 3.339 | 3.546 | -1.204 | 0.230 |
| CGDI | 4.000 | 4.187 | -1.041 | 0.299 |
| Early respondents = first 25% of responses, Late respondents = last 25%, *PP* Proactive personality, *AIJRT* AI job replacement threat, *CA* Challenge appraisal, *HA* Hindrance appraisal, *CGRI* Career goal reengagement intention, CGDI Career goal disengagement intention. | | | | |

**Appendix 4.** Common method bias assessment using the common method factor(Study 2).

| Construct | Indicator | Substantive Factor Loading(*R_1_*) | *R_1_^2^* | Method Factor Loading (*R_2_*) | *R_2_^2^* |
| --- | --- | --- | --- | --- | --- |
| AI job replacement threat(AIJRT) | AIJRT1 | 0.800*** | 0.640 | 0.134* | 0.018 |
|  | AIJRT2 | 0.780*** | 0.608 | 0.138* | 0.019 |
|  | AIJRT3 | 0.857*** | 0.734 | -0.064 | 0.004 |
|  | AIJRT4 | 0.851*** | 0.724 | -0.151** | 0.023 |
|  | AIJRT5 | 0.795*** | 0.632 | -0.034 | 0.001 |
|  | AIJRT6 | 0.799*** | 0.638 | -0.013 | 0.000 |
| Challenge appraisal(CA) | CA1 | 0.865*** | 0.748 | -0.007 | 0.000 |
|  | CA2 | 0.814*** | 0.663 | 0.012 | 0.000 |
|  | CA3 | 0.822*** | 0.676 | 0.028 | 0.001 |
|  | CA4 | 0.865*** | 0.748 | -0.032 | 0.001 |
| Career goal disengagement intention(CGDI) | CGDI1 | 0.754*** | 0.569 | 0.005 | 0.000 |
|  | CGDI2 | 0.863*** | 0.745 | -0.035 | 0.001 |
|  | CGDI3 | 0.864*** | 0.746 | 0.017 | 0.000 |
|  | CGDI4 | 0.782*** | 0.612 | 0.015 | 0.000 |
| Career goal reengagement intention(CGRI) | CGRI1 | 0.790*** | 0.624 | 0.056 | 0.003 |
|  | CGRI2 | 0.844*** | 0.712 | -0.013 | 0.000 |
|  | CGRI3 | 0.865*** | 0.748 | -0.036 | 0.001 |
|  | CGRI4 | 0.857*** | 0.734 | 0.005 | 0.000 |
|  | CGRI5 | 0.794*** | 0.630 | 0.005 | 0.000 |
|  | CGRI6 | 0.673*** | 0.453 | -0.016 | 0.000 |
| Hindrance appraisal(HA) | HA1 | 0.829*** | 0.687 | -0.093* | 0.009 |
|  | HA2 | 0.826*** | 0.682 | 0.062 | 0.004 |
|  | HA3 | 0.834*** | 0.696 | 0.046 | 0.002 |
|  | HA4 | 0.837*** | 0.701 | -0.017 | 0.000 |
| Proactive personality(PP) | PP1 | 0.796*** | 0.634 | -0.016 | 0.000 |
|  | PP2 | 0.804*** | 0.646 | -0.016 | 0.000 |
|  | PP3 | 0.867*** | 0.752 | -0.028 | 0.001 |
|  | PP4 | 0.811*** | 0.658 | 0.016 | 0.000 |
|  | PP5 | 0.860*** | 0.740 | 0.018 | 0.000 |
|  | PP6 | 0.872*** | 0.760 | 0.016 | 0.000 |
|  | PP7 | 0.847*** | 0.717 | 0.013 | 0.000 |
|  | PP8 | 0.793*** | 0.629 | -0.019 | 0.000 |
|  | PP9 | 0.729*** | 0.531 | 0.029 | 0.001 |
|  | PP10 | 0.821*** | 0.674 | -0.011 | 0.000 |
| Average | | 0.819 | 0.673 | 0.000 | 0.003 |
| **p* < 0.05, ***p* < 0.01, ****p* < 0.001 | | | | | |

**Appendix 5.** Analysis results of the marker variable method (Study 2).

| Panel / Path | Before adding marker variable *β* | p-value | After adding marker variable *β* | *p*-value | *Δβ / ΔR²* | Conclusion |
| --- | --- | --- | --- | --- | --- | --- |
| **Panel A. Substantive paths** | | | | | | |
| AIJRT → CA | 0.522 | 0.000 | 0.520 | <0.001 | -0.002 | Stable |
| AIJRT → HA | 0.528 | 0.000 | 0.528 | <0.001 | 0.000 | Stable |
| CA → CGRI | 0.511 | 0.000 | 0.511 | <0.001 | 0.000 | Stable |
| HA → CGDI | 0.554 | 0.000 | 0.554 | <0.001 | 0.000 | Stable |
| PP × AIJRT → CA | 0.187 | 0.000 | 0.183 | <0.001 | -0.004 | Stable |
| PP × AIJRT → HA | -0.032 | 0.410 | -0.031 | 0.425 | +0.001 | Stable |
| AIJRT → CA → CGRI | 0.267 | 0.000 | 0.266 | <0.001 | -0.001 | Stable |
| AIJRT → HA → CGDI | 0.292 | 0.000 | 0.293 | <0.001 | +0.001 | Stable |
| PP × AIJRT → CA → CGRI | 0.096 | 0.000 | 0.093 | <0.001 | -0.003 | Stable |
| PP × AIJRT → HA → CGDI | -0.018 | 0.412 | -0.017 | 0.424 | +0.001 | Stable |
| **Panel B. Marker-variable paths** | | | | | | |
| MV → CA | — | — | -0.073 | 0.297 | — | Not significant |
| MV → HA | — | — | 0.017 | 0.804 | — | Not significant |
| MV → CGRI | — | — | -0.000 | 0.997 | — | Not significant |
| MV → CGDI | — | — | 0.011 | 0.825 | — | Not significant |
| MV → CA → CGRI | — | — | -0.037 | 0.299 | — | Not significant |
| MV → HA → CGDI | — | — | 0.009 | 0.806 | — | Not significant |
| **Panel C. Explanatory power (R²)** | | | | | | |
| CA | 0.310 | — | 0.316 | — | +0.006 | Minimal change |
| HA | 0.280 | — | 0.280 | — | 0.000 | No change |
| CGRI | 0.263 | — | 0.263 | — | 0.000 | No change |
| CGDI | 0.309 | — | 0.309 | — | 0.000 | No change |

*PP* Proactive personality, *AIJRT* AI job replacement threat, *CA* Challenge appraisal, *HA* Hindrance appraisal, *CGRI* Career goal reengagement intention, *CGDI* Career goal disengagement intention.

**Appendix 6.** Assessment of the measurement model(Study2).

| Constructs | Items | Loading | Cronbach’s α | CR | AVE |
| --- | --- | --- | --- | --- | --- |
| AI job replacement threat(AIJRT) | AIJRT1 | 0.808 | 0.898 | 0.922 | 0.663 |
|  | AIJRT2 | 0.788 |  |  |  |
|  | AIJRT3 | 0.850 |  |  |  |
|  | AIJRT4 | 0.840 |  |  |  |
|  | AIJRT5 | 0.794 |  |  |  |
|  | AIJRT6 | 0.801 |  |  |  |
| Challenge appraisal(CA) | CA1 | 0.860 | 0.863 | 0.907 | 0.709 |
|  | CA2 | 0.821 |  |  |  |
|  | CA3 | 0.824 |  |  |  |
|  | CA4 | 0.861 |  |  |  |
| Career goal disengagement(CGDI) | CGDI1 | 0.760 | 0.833 | 0.889 | 0.668 |
|  | CGDI2 | 0.859 |  |  |  |
|  | CGDI3 | 0.861 |  |  |  |
|  | CGDI4 | 0.784 |  |  |  |
| Career goal reengagement intention(CGRI) | CGRI1 | 0.805 | 0.891 | 0.917 | 0.650 |
|  | CGRI2 | 0.844 |  |  |  |
|  | CGRI3 | 0.866 |  |  |  |
|  | CGRI4 | 0.860 |  |  |  |
|  | CGRI5 | 0.786 |  |  |  |
|  | CGRI6 | 0.656 |  |  |  |
| Hindrance appraisal(HA) | HA1 | 0.825 | 0.851 | 0.899 | 0.691 |
|  | HA2 | 0.834 |  |  |  |
|  | HA3 | 0.832 |  |  |  |
|  | HA4 | 0.833 |  |  |  |
| Proactive personality(PP) | PP1 | 0.768 | 0.946 | 0.953 | 0.670 |
|  | PP2 | 0.775 |  |  |  |
|  | PP3 | 0.853 |  |  |  |
|  | PP4 | 0.820 |  |  |  |
|  | PP5 | 0.853 |  |  |  |
|  | PP6 | 0.894 |  |  |  |
|  | PP7 | 0.856 |  |  |  |
|  | PP8 | 0.782 |  |  |  |
|  | PP9 | 0.749 |  |  |  |
|  | PP10 | 0.822 |  |  |  |

**Appendix 7.** The results of HTMT inference (Study 2).

| Set | Original sample (O) | Sample average (M) | 2.5% | 97.5% | Sample average (M) | Bias | 2.5% | 97.5% |
| --- | --- | --- | --- | --- | --- | --- | --- | --- |
| CA ↔ AIJRT | 0.579 | 0.578 | 0.489 | 0.660 | 0.578 | -0.001 | 0.492 | 0.661 |
| CGDI ↔ AIJRT | 0.325 | 0.325 | 0.220 | 0.427 | 0.325 | 0.000 | 0.219 | 0.424 |
| CGDI ↔ CA | 0.256 | 0.256 | 0.158 | 0.357 | 0.256 | 0.000 | 0.158 | 0.356 |
| CGRI ↔ AIJRT | 0.237 | 0.236 | 0.140 | 0.330 | 0.236 | 0.000 | 0.141 | 0.330 |
| CGRI ↔ CA | 0.577 | 0.576 | 0.495 | 0.651 | 0.576 | 0.000 | 0.494 | 0.650 |
| CGRI ↔ CGDI | 0.068 | 0.092 | 0.051 | 0.165 | 0.092 | 0.024 | 0.037 | 0.100 |
| HA ↔ AIJRT | 0.600 | 0.600 | 0.518 | 0.679 | 0.600 | 0.000 | 0.515 | 0.677 |
| HA ↔ CA | 0.262 | 0.263 | 0.166 | 0.359 | 0.263 | 0.001 | 0.164 | 0.357 |
| HA ↔ CGDI | 0.658 | 0.659 | 0.573 | 0.737 | 0.659 | 0.001 | 0.569 | 0.732 |
| HA ↔ CGRI | 0.040 | 0.073 | 0.044 | 0.120 | 0.073 | 0.034 | 0.030 | 0.036 |
| PP ↔ AIJRT | 0.080 | 0.097 | 0.051 | 0.177 | 0.097 | 0.017 | 0.045 | 0.148 |
| PP ↔ CA | 0.083 | 0.098 | 0.049 | 0.181 | 0.098 | 0.014 | 0.045 | 0.160 |
| PP ↔ CGDI | 0.059 | 0.084 | 0.045 | 0.156 | 0.084 | 0.025 | 0.034 | 0.082 |
| PP ↔ CGRI | 0.114 | 0.127 | 0.072 | 0.210 | 0.127 | 0.013 | 0.066 | 0.189 |
| PP ↔ HA | 0.044 | 0.075 | 0.043 | 0.144 | 0.075 | 0.031 | 0.032 | 0.045 |

The HTMT values are all below 0.85, and the confidence intervals do not include 1, indicating that discriminant validity was established for Study 2. *PP* Proactive personality, *AIJRT* AI job replacement threat, *CA* Challenge appraisal, *HA* Hindrance appraisal, *CGRI* Career goal reengagement intention, CGDI Career goal disengagement intention.

**Appendix 8.** Collinearity statistics of the inner model (VIF) (Study2).

| Path | VIF |
| --- | --- |
| AI usage frequency → CGDI | 1.001 |
| AI usage frequency → CGRI | 1.001 |
| AIJRT → CA | 1.005 |
| AIJRT → HA | 1.005 |
| Age → CGDI | 1.004 |
| Age → CGRI | 1.004 |
| CA → CGRI | 1.002 |
| HA → CGDI | 1.001 |
| PP → CA | 1.006 |
| PP → HA | 1.006 |
| Gender → CGDI | 1.004 |
| Gender → CGRI | 1.006 |
| PP x AIJRT → CA | 1.000 |
| PP x AIJRT → HA | 1.000 |

*PP* Proactive personality, *AIJRT* AI job replacement threat, *CA* Challenge appraisal, *HA* Hindrance appraisal, *CGRI* Career goal reengagement intention, CGDI Career goal disengagement intention.

## **Appendix 9**. CVPAT results (Study 2).

| Construct | PLS loss | IA loss | Average loss difference | *t* value | *p* value |
| --- | --- | --- | --- | --- | --- |
| CA | 2.533 | 3.212 | -0.679 | 7.030 | <0.001 |
| CGDI | 2.568 | 2.704 | -0.136 | 2.820 | 0.005 |
| CGRI | 2.603 | 2.715 | -0.111 | 2.467 | 0.014 |
| HA | 2.442 | 2.996 | -0.554 | 6.333 | <0.001 |
| Overall | 2.544 | 2.885 | -0.342 | 8.223 | <0.001 |

*PP* Proactive personality, *AIJRT* AI job replacement threat, *CA* Challenge appraisal, *HA* Hindrance appraisal, *CGRI* Career goal reengagement intention, CGDI Career goal disengagement intention.
